# Gamma Knife radiosurgery for locally recurrent choroidal melanoma following plaque radiotherapy

**DOI:** 10.1186/s40942-018-0123-1

**Published:** 2018-06-13

**Authors:** Osama A. Sorour, John E. Mignano, Jay S. Duker

**Affiliations:** 1 New England Eye Center, Department of Radiation Oncology, Tufts Medical Center, Tufts University School of Medicine, Boston, MA USA; 2 Ophthalmology Department, Faculty of Medicine, Tanta University, Tanta, Egypt

**Keywords:** Melanoma, Choroidal, Uveal, Brachytherapy, Gamma Knife, Tumor, Ocular, Radiosurgery, Radiation

## Abstract

**Background:**

For the majority of eyes with choroidal melanoma, radiation therapy is the treatment of choice. Local recurrence after radiation therapy can occur, however, and when it does, salvaging the globe with useful vision is atypical.

**Case presentation:**

We report a case of late, local failure 7 years following previous brachytherapy successfully managed with Gamma Knife radiosurgery (GKR). With 3 years of follow up after GKR, the visual acuity is 20/20 and there is no evidence of systemic metastases.

**Conclusion:**

To our knowledge, this is the first report of successful salvage GKR therapy after brachytherapy failure in an eye with choroidal melanoma. GKR is an option for select cases of local recurrence after radiation plaque brachytherapy.

## Background

Choroidal melanoma, although rare, is the most common primary intraocular malignancy in adults with reported incidence of 5.1 per million people [1]. The tumors usually presents as a dome-shaped mass with a smooth surface. If there is tumor growth through Bruch’s membrane a mushroom configuration can be observed. Choroidal melanoma is usually pigmented (55%), but may be nonpigmented (15%), or exhibit mixed color (30%). The diagnosis is largely based on clinical examination and ancillary testing. Occasionally biopsy is used. Serial examinations with ancillary testing to confirm growth may be necessary in some cases [2].

Ultrasonography plays an important role both in diagnosis and follow up of the patient. Features include acoustic hollowing, choroidal excavation and orbital shadowing [3]. Other imaging techniques like fluorescein angiography (FA) and indocyanine green angiography (ICG) are of limited value in management of choroidal melanoma. A ‘double circulation’ pattern may be seen in tumors infiltrating through Bruch’s membrane with FA [4]. ICG offers better visualization of melanoma vasculature and may help in differentiating choroidal melanoma from hemangioma in certain situations [5].

Worldwide, plaque radiotherapy (brachytherapy) is the most common treatment for choroidal melanoma. Various isotopes have been used for brachytherapy, the most common are the gamma emitting radionuclides; iodine-125, cesium-131, and palladium-103. Ruthenium-106, which emits β-particles, can be used as well however its limited tissue penetration of 4–5 mm; makes it suitable only for tumors less than 5 mm in height [6].

Stereotactic radiation therapy using Cyber Knife, Gamma Knife, or linear accelerator as well as charged particles (protons) have been used successfully in the management of choroidal melanoma. Results of overall survival, visual outcome, and local tumor control, in patients received stereotactic radiation appear equivalent to those undergoing plaque radiotherapy [7, 8].

## Case presentation

**Fig. 1:**
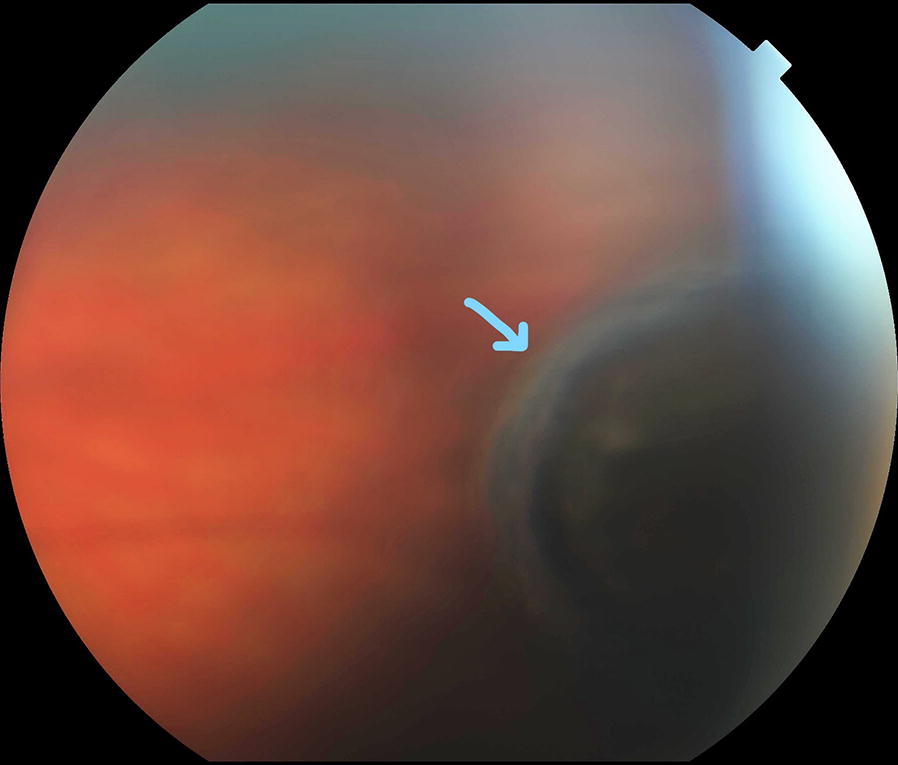
Color fundus photography of the patient at initial presentation in September 2007, showing far peripheral pigmented choroidal mass (blue arrow), that revealed to be melanoma

A female patient aged 36 years was seen initially in September of 2007, complaining of floaters in her right eye. Examination revealed a choroidal pigmented peripheral mass that measured 2.9 mm in thickness on ultrasonography (Fig. 1). Observation was recommended. At 3 months follow up, the lesion had increased to 3.8 mm in thickness, and the diagnosis of choroidal melanoma was made. Systemic evaluation was normal In December 2007 she underwent treatment via low-dose-rate temporary plaque brachytherapy. A 16 mm Choroidal Ocular Melanoma Study (COMS) applicator was loaded with 13 cesium-131 sources and was used to deliver a dose of 85 gray (Gy) to a prescription height of 5 mm, over an implant duration of 117.5 h.

Three years later, after uncomplicated cataract surgery, her visual acuity was 20/20, there was no evidence of radiation retinopathy and the lesion appeared regressed with a thickness of 3.1 mm via ultrasonography.

**Fig. 2:**
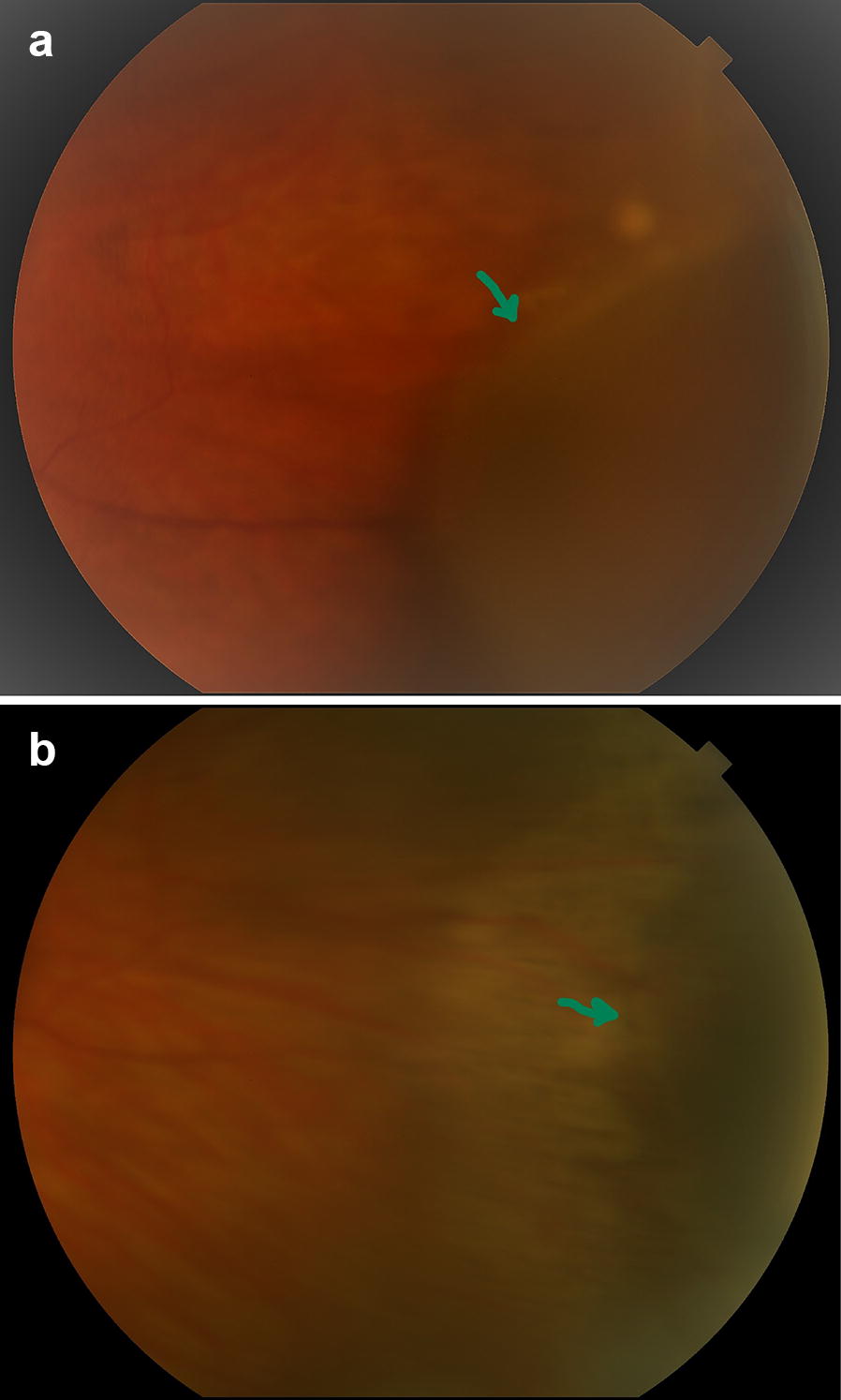
Color fundus photography of the patient **a** immediately before GK radiosurgery the lesion has increased in basal thickness and appears enlarging and to extend beyond the previous line of stabilization, and **b** in December 2017 the tumor continued to regress after the GKR, note the green arrow illustrating change from active fleshy tumor to atrophic one

**Fig. 3:**
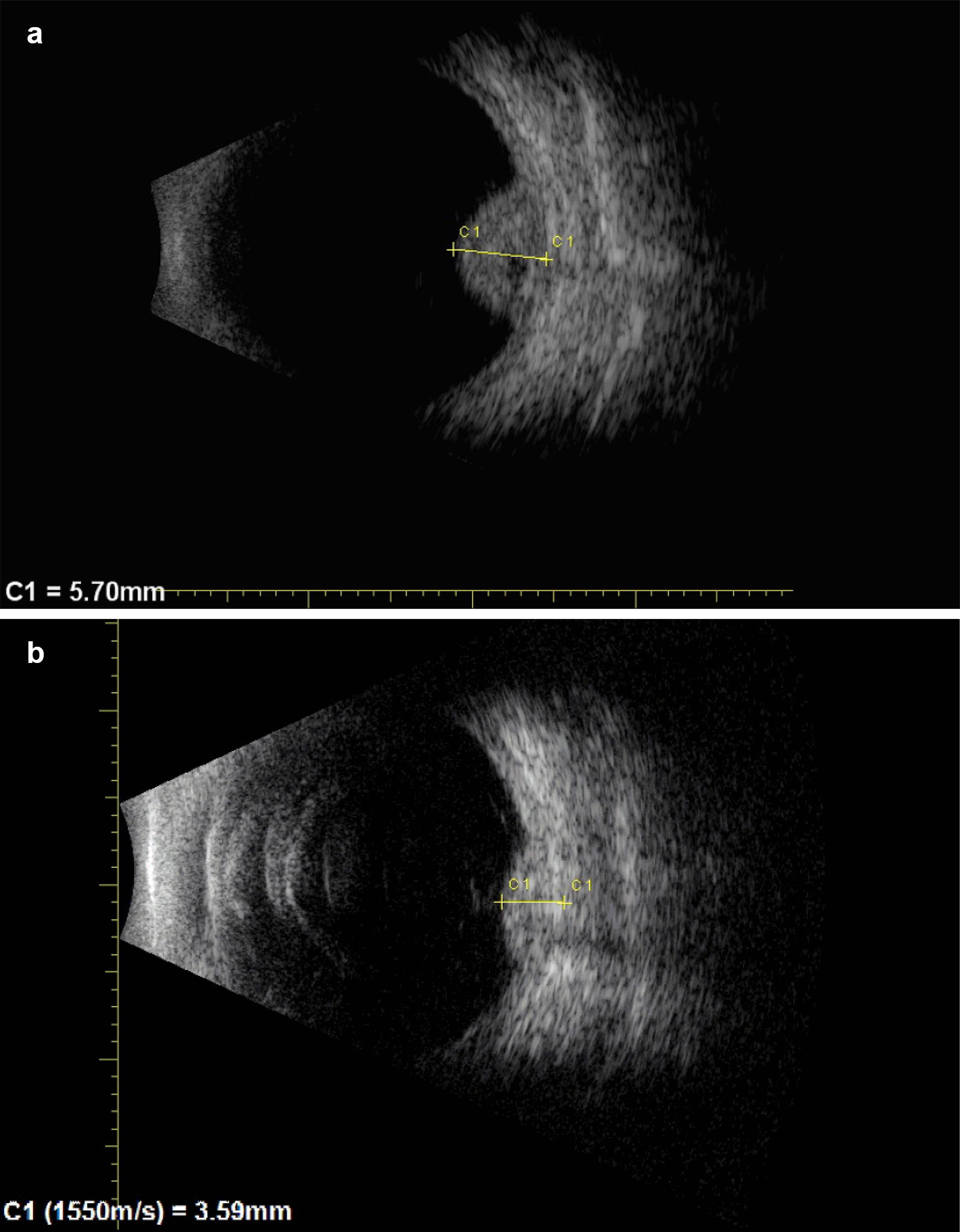
Ultrasonography of the patient **a** immediately before GK radiosurgery: tumor has increased in thickness to 5.7 mm after years of stabilization, and **b** in October 2017 the tumor has successfully shrunk after GKR to be 3.59 mm

Seven years after brachytherapy, on routine yearly follow-up examination, the thickness of the tumor measured by ultrasound appeared to increase to 3.99 mm. As she was pregnant at the time, it was decided to observe the lesion. Following delivery of a healthy baby, and subsequent confirmed growth to a thickness of 5.7 mm, a fine needle aspiration biopsy was performed (Figs. 2a and 3a).

Cytology was positive for choroidal melanoma and genetic profile (Castle Biosciences) test revealed a Class 2 tumor. A restaging evaluation was completed and was negative for metastatic disease. As the patient had excellent vision in the treated eye, and the lesion was peripheral, a decision to proceed with re-irradiation via GKR as opposed to enucleation was made.

GRK was performed in March 2015. The retrobulbar/peribulbar nerve block was performed in standard fashion as was placement of the stereotactic headframe. For target localization, T1 contrast enhanced and T2 weighted MRI sequences were obtained as per routine for GRK treatment planning. At that time the melanoma volume was ~ 0.2 mL and the radiosurgery delivered a dose of 20 Gy to the 50% isodose line via a plan consisting of 3 isocenters of 4 mm collimation and 2 isocenters of 8 mm collimation. Custom sector blocking was utilized to increase the isodose conformality. With this treatment plan the minimum peripheral tumor dose was 21.75 Gy and the maximum point dose within the optic nerve was 4 Gy. This dose was well within generally accepted limits for the optic nerve, even considering the previously received scatter dose from the patient’s brachytherapy.

Follow-up revealed excellent early results with decrease of tumor size to 3.79 after 4 months. At the last follow-up visit, 3 years after GKR, the visual acuity remained 20/20, and tumor thickness was 3.31 mm with stable lesion borders. There was no radiation retinopathy nor evidence of systemic metastases (Figs. 2b and 3b).

## Discussion and conclusions

Since radiation therapies, such as brachytherapy, appear to offer the same survival outcome versus enucleation for medium-sized choroidal melanomas (i.e. with basal diameter < 16 mm and thickness 2.5–10 mm), they are the first treatment choice in most cases [9, 10].

GKR was first applied in the treatment of brain tumors. This technique delivers a single dose of ionizing radiation to a relatively small target with a steep decrease of dose at the margins. Various studies suggest GKR has similar efficacy to proton beam therapy and plaque brachytherapy for the treatment of choroidal melanoma [11, 12]. However, this technique has been associated with high reported rates of radiation retinopathy and neovascular glaucoma [13, 14]. Appropriate ocular fixation represents some challenge to this technique. Higher ocular morbidity was reported in treating melanoma larger than 8 mm thick. Larger dose than 10 Gy/fraction were associated with greater risk radiation-induced inflammation [15].

In the case reported herein, our patient has a base thickness of choroidal melanoma of 5.71, and dose-planning took in consideration the previous radiation treatment to deliver the optimum dose of radiation and lessen potential hazards. The minimum peripheral tumor dose was 21.75 Gy and the maximum point dose within the optic nerve was 4 Gy [16].

Although local treatment failure is a recognized complication of choroidal melanoma radiation treatment, few publications have addressed this issue. Patients with local recurrence may have an increased risk of metastasis and decreased survival [17]. Moreover, re-treatment options of those patients are limited. Some local recurrences can be treated with focal laser, cryotherapy, or laser hyperthermia but the majority undergo enucleation [18].

The reported rates of local recurrence vary depending on tumor location and thickness. In a large study comprising 650 patients, and follow up of 60 months after iodine-125 brachytherapy the local failure rate was 10.3% [19]. Local recurrence rate of 9% was reported in 75 patients undergoing GKR [14]. In a meta-analysis of local treatment failures across various centers, it was found that radiation therapy was superior to surgical and laser modalities of treatment for achieving local tumor control. Weighted mean failure rates were 6.15% for radiation, 18.6% for surgical, and 20.8% for laser treatments, also, there was no difference in failure rates between various isotopes used for brachytherapy. The rate of local treatment failure with GKR appears similar to that of brachytherapy, however, the risk of radiation-induced side effects may be higher [18]. In our case the tumor’s initial thickness was less than 4 mm which would make the incidence of recurrence less likely, however there was local tumor recurrence 7 years after brachytherapy.

In the presented case GKR was used as an eye preserving salvage therapy after late local recurrence of ocular melanoma. With 10 years of follow up after initial diagnosis there is no sign of systemic metastases. Despite two radiation therapies and cataract surgery, the vision in the treated eye remains 20/20. We believe this is the first successful case of GKR for local failure after brachytherapy. GKR may be an option for select cases of local recurrence after radiation plaque brachytherapy.

## Abbreviations

EDI-OCT: enhanced depth imaging optical coherence tomography
FA: fluorescein angiography
ICG: indocyanine green angiography
GKR: Gamma Knife radiosurgery
Gy: gray, which is the unit of ionizing radiation by the international system of units
COMS: Choroidal Ocular Melanoma Study
